# Treatment of an adrenomyeloneuropathy patient with Lorenzo's oil and supplementation with docosahexaenoic acid-A case report

**DOI:** 10.1186/1476-511X-10-152

**Published:** 2011-08-26

**Authors:** Gisella Terre'Blanche, Mietha M van der Walt, Jacobus J Bergh, Lodewyk J Mienie

**Affiliations:** 1 Pharmaceutical Chemistry, Unit for Drug Research and Development, School of Pharmacy, North-West University, Private Bag X6001, Potchefstroom, 2520, South Africa; 2 Biochemistry, School of Physical and Chemical Sciences, North-West University, Private Bag X6001, Potchefstroom 2520, South Africa

**Keywords:** Adrenoleukodystrophy, Adrenomyeloneuropathy, Lorenzo's oil, Docosahexaenoic acid, polyunsaturated fatty acids

## Abstract

This is a case report of adrenomyeloneuropathy (AMN), the adult variant of adrenoleukodystryphy (ALD). The diagnoses in the patient, aged 34, was confirmed via increased serum very long chain fatty acid concentration (VLCFA). Treatment started with the cholesterol lowering drug, atorvastatin, followed by add-on therapy with Lorenzo's oil (LO) and finally supplementation with docosahexaenoic acid (DHA). The magnetic resonance imaging (MRI) scan of the AMN patient before DHA treatment, already showed abnormal white matter in the brain. Although the MRI showed no neurological improvement after 6 months of DHA treatment, no selective progression of demyelination was detected in the AMN patient. Contrary to what was expected, LO failed to sustain or normalize the VLCFA levels or improve clinical symptoms. It was however, shown that DHA supplementation in addition to LO, increased DHA levels in both plasma and red blood cells (RBC). Additionally, the study showed evidence that the elongase activity in the elongation of eicosapentaenoic acid (EPA) to docosapentaenoic acid (DPA) might have been significantly compromised, due to the increased DHA levels.

## Introduction

Adrenomyeloneuropathy (AMN), one of the variants of X-linked adrenoleukodystrophy (X-ALD), is an inherited genetic disorder, classified as a single peroxisomal enzyme disorder that affects the peroxisomal β-oxidation pathway. Cerebral demyelination may develop in males and is confirmed via clinical and MRI evidence of inflammatory brain involvement [1]. Biochemically, this disease is associated with the abnormal accumulation of saturated, very long chain fatty acids (VLCFA), especially C24:0 and C26:0 [2].

Although different therapies have been employed to treat X-ALD, there is currently no cure. Recent articles suggest cholesterol management as a potential treatment. In X-ALD patients, accumulation of VLCFA is mainly in the form of cholesterol ester fractions in ALD tissues, particularly the brain [3]. It was demonstrated in a previous study that the HMG-CoA reductase inhibitor, lovastatin, caused a reduction in the VLCFA plasma levels of X-ALD patients [4]. Therefore, it is contemplated that an interaction between cholesterol and VLCFA metabolism does exist [5]. However, the exact mechanism is still unknown. Another alternative therapy consists of Lorenzo's oil (LO) (mixture of oleic and erucic acid) in combination with a diet low in VLCFA. Although VLCFA plasma levels of ALD patients were lowered within 4 weeks after treatment with LO [6], no improvement of neurological symptoms has been reported in the literature [7]. Elevated levels of erucic and nervonic acids in plasma, as well as a reduction in docosahexaenoic acid (DHA) and eicosapentanoic acid (EPA) had been observed in patients treated with LO [8]. Results from another study also indicated a decrease in DHA plasma levels after treating AMN patients with LO [9], leading to speculation that erucic acid could have an effect on the metabolism of DHA. The mechanism of action of Lorenzo's oil could be attributed to erucic acid that competes for the elongation of saturated fatty acids in the VLCFA synthesis pathway, resulting in a reduction of the VLCFA concentrations [8]. Competition for the elongation enzyme in the DHA biosynthesis pathway by erucic acid could be a possible mechanism for decreased DHA levels observed with Lorenzo's oil treatment. In order to normalize the essential fatty acid depletion, supplementation with DHA is usually recommended [8].

The aim of this study, was to establish if supplementation with DHA in addition to treatment with LO, might increase plasma and red blood cell (RBC) DHA levels and improve clinical symptoms in a patient diagnosed with X-ALD (phenotype AMN).

### Clinical Report

A 28-year-old male patient started to complain about finding it difficult to walk as well as of bladder dysfunction. Magnetic Resonance Imaging (MRI) confirmed the diagnosis of a degenerative neurological disorder. At age 34, the diagnoses of ALD was confirmed at the laboratory for Inherited Metabolic Defects, School for Biochemistry, North-West University (Potchefstroom Campus), South Africa, as well as at the laboratory for Genetische Metabole Ziekten in Amsterdam, The Netherlands, via increased plasma levels of VLCFA (C26:0 and C24:0) as well as high ratios of C24:0/C22:0 and C26:0/C22:0.

The clinical picture was typical of the AMN phenotype. The following symptoms were present during physical examination: spastic paraparesis, ataxia, variable episodes of hypertonia and spasms, impotence (possibly related to the spinal cord involvement), intermittent behavioural changes and bladder dysfunction.

Approximately one year after diagnosis, brain involvement was again assessed by MRI. Even though abnormal white matter was clearly observed, the patient was diagnosed with the AMN phenotype. At that time, the neurological progression of the patient was also rated using the ALD-Disability Rating Scale [10]. On that occasion, the patient was rated at number two, indicating that he did require some interventions. No adrenal insufficiency was noted with an ACTH stimulation test.

Treatment with the cholesterol lowering drug, atorvastatin (10 mg/day), and L-carnitine (2g/day) started immediately after diagnosis. After three months, LO (10 ml/day) was added to the treatment to effect inhibition of VLCFA elongation. This treatment continued for seven months (March 2008 to September 2008) and was then supplemented with DHA (600 mg/day in divided doses) for eight months. The DHA supplementation consisted of a mixture of medium chain triglyceride oil (MCT), fish oil (40% DHA, 5% EPA) and vitamin E as an antioxidant.

The VLCFA, phytanic and pristanic acid content in plasma, during all the treatment phases (cholesterol lowering, LO, DHA) and the polyunsaturated fatty acid (PUFA) content in plasma during LO treatment were determined. The effect of the DHA treatment was established by determining PUFA in both plasma and RBC and a clinical evaluation was carried out using an ALD-Disability Rating Scale and an MRI scan. The latter evaluations were done before and after DHA therapy started. Plasma concentrations of oleic and erucic acid were measured every month to corroborate compliance to the dietary treatment.

### Analytical Procedures

This case study was approved by the Ethics Committee of the North-West University (Potchefstroom Campus) (application number NWU-0038-08-S5). Informed consent was obtained from the patient for this case study.

#### Blood collection

The AMN patient fasted for 10 to 14 hours prior to the collection of blood samples. For analysis of the PUFA, blood samples were collected in vacutainers containing EDTA.

The method described by Evans and co-workers [11], was used for separating plasma and RBC and samples were stored at -80°C until analysis. Plasma and RBC were obtained from age matched healthy human subjects.

#### Analysis of saturated VLCFAs, in plasma

A modified method from Vreken and co-workers [12] was used for sample preparation and determination of VLCFAs. The samples were analyzed by GC/MS (Hewlett-Packard model 6890/5973 GC-MS system), equipped with a 120-0132 DB-1ms capillary column (30 m × 0.25 mm i.d. × 0.25 μm film thickness) (Agilent Technologies, Chemetrix, Midrand, South Africa).

Samples were injected into the GC/MS system with splitless mode and the carrier gas was helium. The electron impact ionization was applied at 0.7 eV and the mass spectrum with single ion monitoring (SIM) mode, was used to monitor the characteristic [M-57]^+ ^ions [12]. The oven temperature was programmed to start at a temperature of 60°C for 1 minute. The temperature was then increased to 240°C (30°C/min), followed by an increase to 270°C (10°C/min) and finally by an increase to 300°C (4°C/min) where it was maintained for 3 minutes. HP-chemstation software was used to quantify the raw data obtained from the GC/MS.

#### Analysis of PUFA in RBC

For the sample preparation of the RBC a modified version of the procedures by Takemoto and co-workers [2] and by Blau and co-workers [13] were used. The concentrations of the fatty acid methyl esters (FAME) in the RBC samples were analyzed by GC/MS (Agilent Technologies 6890N GC system and Agilent Technologies 5973 MS system) equipped with a capillary column (Agilent 19091S-433). The injector (7683 B Series) temperature was set at 250°C and helium was used as carrier gas. The RBC samples were injected into the GC/MS system with splitless mode. The oven temperature was initially set at 50°C and was increased to 190°C (30°C/min), then to 220°C (3°C/min), and finally to 230°C (6°C/min) and maintained for 24 minutes.

Identification of the PUFA peaks was carried out via AMDIS, an Automated Mass Spectral Deconvolution and Identification System. The mass spectra of the fatty acids were obtained from the National Institute of Standards and Technology (NIST) library.

#### Analysis of PUFA in plasma with TLC

The methods of Takemoto and colleagues [2] and Blau and co-workers [13], were modified for sample preparation. Purification of the samples was achieved by adapting the thin-layer chromatography method by Smuts and Tichelaar [14] for methyl esters. The samples were analyzed with a GC/MS (Agilent Technologies 6890N GC system and Agilent Technologies 5973 MS system) equipped with a capillary column (J&W 122-2361). The injector (7683 B Series) temperature was set at 250°C and helium was used as carrier gas.

The plasma samples were injected into the GC/MS system with split mode. The split ratio was set at 5:1 with a flow of 4.4 ml/min. The oven temperature was initially set to 160°C and increased to 220°C (3°C/min) where it was maintained for 15 minutes. Samples were quantified with Chemstation Enhanced Data analysis software (version D.01.XX) using unique single ions and calibration curves for each FAME.

## Results and discussion

**Figure 1:**
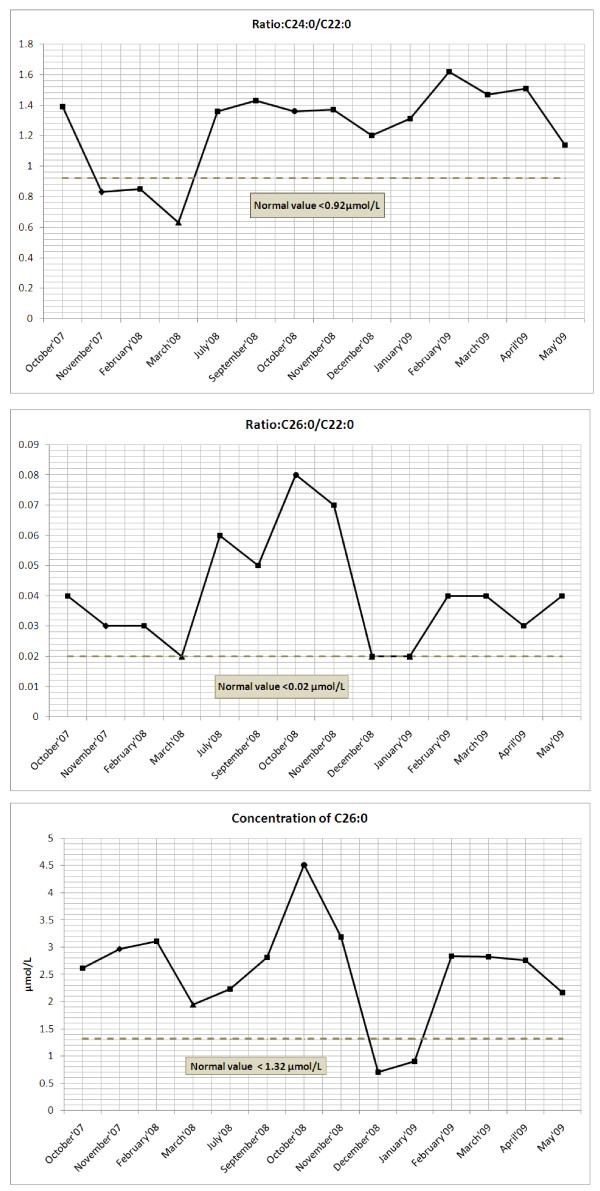
**VLCFA plasma levels of the AMN patient: where (a) depicts the ratio C24:0/C22:0; (b) depicts the ratio C26:0/C22:0 and (c) depticts the concentration C26:0**. The symbols represent: black diamond-one month after artorvastatin and L-carnitine started (November 2007); black triangle-one month after Lorenzo's oil started (March 2008) and the black sircle-one month after DHA started (October 2008). The grey dashed line indicates the threshold for the analysed data to be regarded as normal subject values.

The results showed that both the C24:0/C22:0 and C26:0/C22:0 plasma ratios were reduced within one month of starting therapy with atorvastatin and L-carnitine, compared to the pre-treatment value and that these ratios remained lowered during the first three months of treatment (Figure 1a and 1b). Unfortunately, plasma C26:0 concentrations increased slightly (Figure 1c). Currently, the exact mechanism of how cholesterol lowering drugs reduces plasma levels of VLCFA in X-ALD patients is unknown [4]. It has been suggested that the effect of lovastatin on plasma VLCFA may rather be attributed to its effect on the cholesterol synthesis than to a direct effect on the VLCFA metabolism [15]. Since no clinical improvement was noted, LO was added to the treatment regime.

**Figure 2:**
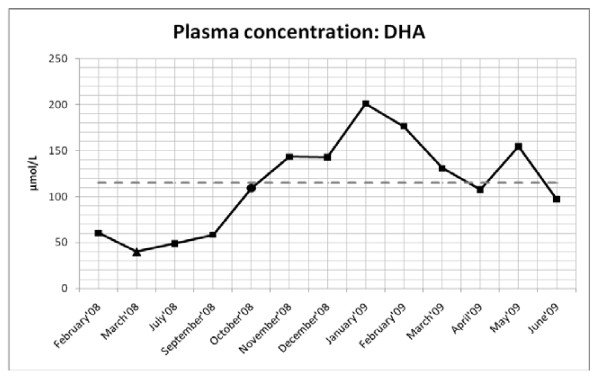
**DHA plasma analysis of the AMN patient where the concentration of DHA is depicted for the period February 2008 to June 2009**. The symbols represent: black triangle-one month after Lorenzo's oil started (March 2008); and the black sircle-one month after DHA started (October 2008). The grey dashed line indicates the average of control samples collected.

Lorenzo's oil decreased plasma VLCFA ratios C24:0/C22:0 and C26:0/C22:0 after one month of treatment, however, over a period of six months after therapy started, these ratios increased to values higher than before LO treatment began (Figure 1a and 1b). Plasma C26:0 concentrations sharply decreased after one month of LO treatment, but levels then started to increase (Figure 1c). Decreased plasma DHA levels (Figure 2), below normal control values, were noted for the entire period in which LO, atorvastatin and L-carnitine were administered (March 2008 to September 2008). Once again it could be argued that reduced levels could be attributed to LO treatment, which is in accordance to the study of Moser and coworkers [16] where they found the severe reduction in ω-3 and ω-6 fatty acids, including DHA during LO treatment in the absence of ω-3 and ω-6 supplementation. In this case study, LO initially decreased plasma VLCFA levels and ratios, but failed to sustain or normalize VLCFA levels and to improve clinical symptoms. We found raised concentrations of oleic and erucic acid in the plasma of the patient that corroborated the adherence to the therapy.

**Figure 3:**
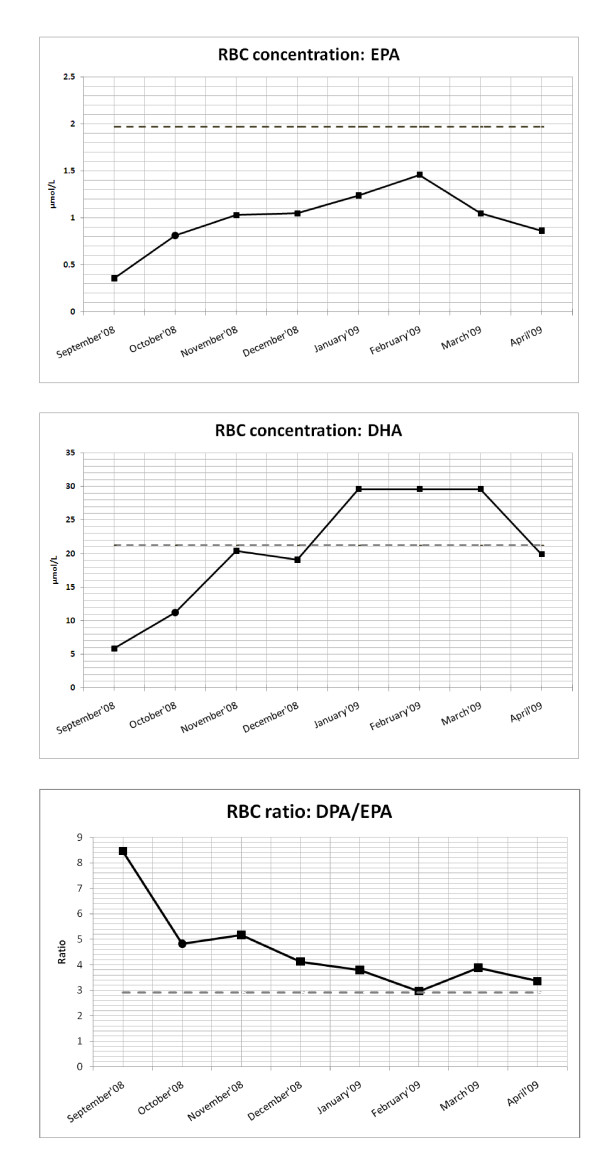
**Omega 3 PUFA RBC analysis of the AMN patient where (a) depicts the concentrations of EPA; (b) depicts the concentrations of DHA and (c) the ratio of DPA/EPA for the period of September 2008 to April 2009**. The black circle, (October 2008), represents one month after DHA supplementation was started. The grey dashed line indicates the average value of control samples collected.

DHA supplementation had no effect on plasma C24:0/C22:0 ratios for the entire therapeutic period (Figure 1a). An unexplained increase in C26:0/C22:0 ratio and C26:0 concentration, one month after DHA therapy started, was noticed, which gradually decreased to normal values in the following three months (November'08 to January'09). Thereafter these values increased, and remained at these higher levels during the DHA treatment phase without any further increase (Figure 1b and 1c). Significantly, supplementation with DHA increased plasma and RBC DHA values (see Figure 2 and 3a) within a few weeks after the initiation of treatment and remained above the mean DHA levels of the control samples collected for a period of six and three months. Hereafter, both plasma and RBC DHA values returned to the mean DHA levels of the control samples.

**Figure 4:**
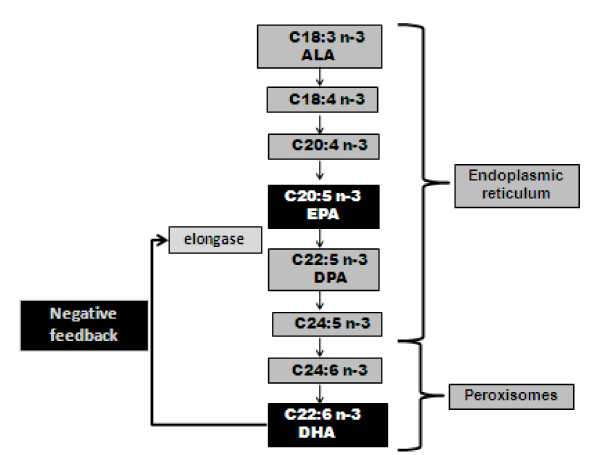
**Schematic of the possible negative feedback mechanism by DHA through inhibition of the elongase between EPA and DPA**. Abreviations: ALA = α-Linoleic acid, EPA = eicosapentaenoic acid DPA = docosapentaenoic acid and DHA = docosahexaenoic acid.

One month after DHA supplementation started, a sharp increase in RBC EPA levels (Figure 3b) was noted, followed by a gradual increase for the remainder of DHA treatment. Coinciding with EPA increasing, the RBC DPA/EPA ratio (Figure 3c) showed a sharp decrease, gradually declining for the remainder of DHA treatment. One mechanism that might account for this decreased DPA/EPA ratio, involves a regulatory mechanism in which the high DHA levels exert a negative feedback mechanism on the elongase enzyme responsible for the elongation of EPA to DPA (Figure 4). The decreased activity of this elongase enzyme subsequently leads to higher EPA levels, thus explaining the increased EPA levels in the patient after DHA supplementation. Supporting these findings is a metabolic study done in 2002 [17], which showed evidence that elongase activities might be altered with long-chain polyunsatured fatty acid feeding. Mice were fed control diets and fish oil enriched with DHA and EPA. The ratios of C22:4 n-6/C20:4 n-6 and C22:5 n-3/C20:5 n-3 were all reduced by the fish oil feeding. The current study also showed a decrease C22:4 n-6/C20:4 n-6 ratio in both plasma and RBC, confirming elongation inhibition (data not shown).

Subsequently, the increased DHA and EPA levels could also lead to an anti-inflammatory effect. DHA and EPA competitively inhibit the oxygenation of arachidonic acid (C20:4 n-6; AA) [18], thereby decreasing production of AA-derived eicosanoids. DHA reduces C20:4 n-6 biosynthesis via the inhibition of Δ5 or Δ6 desaturase [19], whereas EPA displaces C20:4 n-6 from phospholipids. The present study also confirmed the inhibition of Δ6 desaturase, as is indicated by the decreased plasma ratio, C18:3 n-6/C18:2 n-6, after one month of DHA supplementation (data not shown).

**Figure 5:**
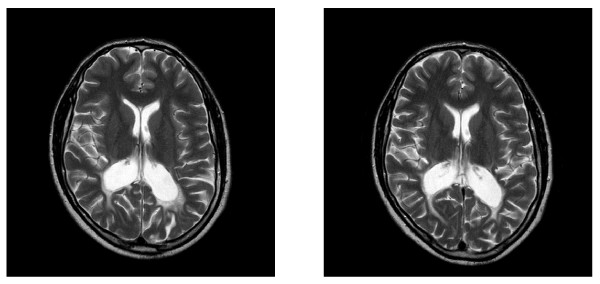
**Brain MRI scans of the AMN patient: T2 weighted one month before DHA treatment began (September 2008) (left) and T2 weighted six months after DHA treatment began (March 2009) (right)**.

The MRI scans illustrate that the AMN patient already showed abnormal white matter lesions, typical of ALD, at the onset of this study. Abnormal white matter was shown on the white matter MRI series, grey matter MRI series, the FLAIR study and the T2 weighted study (Figure 5a and 5b). No definite abnormal white matter progression could be identified on either the FLAIR or the T2 weighted studies for September 2008 and March 2009, during treatment with LO and DHA supplementation. Although the MRI scans before and after DHA supplementation showed no neurological improvement after six months of DHA treatment, no selective progression of demyelination was detected. This stabilization was further supported by the ALD-Disability Rating Scale, that remained unchanged (at number two) for the DHA treatment phase. Since the patient had been diagnosed in October 2007 and had already been showing symptoms when DHA supplementation started a year later, it could be argued that DHA prevented further neurological progression although no improvement was evident.

In conclusion: it was confirmed that supplementation with DHA, in addition to LO and treatment with a cholesterol lowering drug, atorvastatin, increased DHA levels in plasma and RBC of the AMN patient. These increased DHA levels may exert a neuro-protective effect by a negative feedback mechanism, leading to an increase in EPA. In turn, EPA and DHA are incorporated into inflammatory cell phospholipids, partly at the expense of AA, exerting an anti-inflammatory effect [20]. Sing and Pujol also suggested that treatment strategies should be developed for the inflammatory, metabolic and oxidative stress disease aspects of X-ALD [21]. Supplementation with DHA, is therefore strongly recommended in patients with X-ALD patients, due to the important role of DHA in brain development and myelination and the feedback mechanism which may cause a neuro-protective and anti-inflammatory effect.

## Abbreviations

AMN: adrenomyeloneuroophathy; ALD: adrenoleukodystrophy; VLCFA: very long chain fatty acid; LO: Lorenzo's oil; DHA: docosahexaenoic acid; MRI; magnetic resonance imaging; EPA: eicosapentaenoic acid; DPA: docosapentaenoic acid; RBC: red blood cell; PUFA: polyunsaturated fatty acid; FAME: fatty acid methyl esters

## Competing interests

The authors declare that they have no competing interests.

## Authors' contributions

GT designed and coordinated the study, participated in the fatty acid analysis and drafted the manuscript. MMvdW carried out the blood collection and analysis of fatty acids, and assisted in the draft of the manuscript. JJB assisted in the draft of the manuscript and participated in the design of the study. LJM conceived of the study, and participated in its design and coordination.

All authors read and approved the final manuscript.
